# First detection of *Eimeria* species in Myanmar domestic goats with both microscopic and molecular methods

**DOI:** 10.1051/parasite/2020037

**Published:** 2020-05-19

**Authors:** Saw Bawm, Tay Zar Bhone Win, Shwe Yee Win, Lat Lat Htun, Ryo Nakao, Ken Katakura

**Affiliations:** 1 Department of Pharmacology and Parasitology, University of Veterinary Science Yezin 15013 Nay Pyi Taw Myanmar; 2 Department of International Relations and Information Technology, University of Veterinary Science Yezin 15013 Nay Pyi Taw Myanmar; 3 Laboratory of Parasitology, Faculty of Veterinary Medicine, Hokkaido University 060-0818 Sapporo Japan

**Keywords:** *Eimeria arloingi*, *Eimeria hirci*, *Eimeria christenseni*, Domesticated goats, Myanmar

## Abstract

Coccidiosis is of great economic importance in many farm animals. This study involved analysis of 280 faecal samples collected from 12 traditional goat farms from Nay Pyi Taw area, Myanmar. Faecal samples were examined by the flotation method and concentrated oocysts were identified on the basis of morphological characters. Of 280 faecal samples examined, 168 (60.0%) were positive for *Eimeria* oocysts. Three different *Eimeria* species were identified and their positive detection rates in the herd were: *E. arloingi* (25.4%), followed by *E. hirci* (20.7%) and *E. christenseni* (13.9%). Identifications were confirmed by 18S rDNA and COI sequences. 18S rDNA sequences showed 100% homology with, respectively, *E. christenseni* reported from Australia, *E. arloingi* reported from Australia and Iran, and *E. hirci* from Australia. COI sequences of *E. christenseni*, *E. hirci*, and *E. arloingi*, respectively, exhibited 98.9%, 98.4%, and 98.5% similarities with those reported from Australia. This is the first report of *Eimeria* infection in Myanmar goats.

## Abstract (fr)

La coccidiose est d’une grande importance économique pour de nombreux animaux d’élevage. L’étude a impliqué l’analyse de 280 échantillons de matières fécales prélevés dans 12 fermes caprines traditionnelles de la région de Nay Pyi Taw, Myanmar. Les échantillons fécaux ont été examinés par la méthode de flottation et des oocystes concentrés ont été identifiés sur la base de caractères morphologiques. Sur 280 échantillons fécaux examinés, 168 (60,0 %) étaient positifs pour les oocystes d’*Eimeria*. Trois espèces différentes d’*Eimeria* ont été identifiées et leurs taux de détection positifs dans le troupeau étaient: *E. arloingi* (25,4 %), suivi par *E. hirci* (20,7 %) et *E. christenseni* (13,9 %). Les identifications ont été confirmées par les séquences d’ADNr 18S et de COI. Les séquences d’ADNr 18S ont montré une homologie de 100 % avec, respectivement, *E. christenseni* d’Australie, *E. arloingi* d’Australie et d’Iran et *E. hirci* d’Australie. Les séquences de COI d’*E. christenseni*, *E. hirci* et *E. arloingi* présentaient des similitudes de 98,9 %, 98,4 % et 98,5 %, respectivement, avec celles rapportées d’Australie. Il s’agit du premier signalement d’infection à *Eimeria* chez des chèvres du Myanmar.

## Introduction

Coccidiosis of farm animals is caused by coccidial parasites of the genus *Eimeria* which develop in the small and the large intestines. Coccidiosis is of great economic importance in many farm animals, especially young animals, because of losses due to clinical disease (diarrhoea) and subclinical infections (poor weight gain in particular) and the required treatment costs. Many *Eimeria* infections in goats are asymptomatic; however, some species have been associated with diarrhoea and stunted growth [6, 33]. Of the 16 *Eimeria* species identified in goats worldwide, *E. arloingi*, *E. ninakohlyakimovae*, *E. christenseni*, and *E. caprina* were considered to be the most pathogenic species [14, 18, 29, 35]. According to reports from Sri Lanka, Malaysia, China, and India, the most frequent species of *Eimeria* in goats in Asian countries are *E. ninakohlyakimovae*, *E. christenseni*, *E. arloingi*, *E. parva*, *E. caprina*, and *E. alijevi* [11, 14, 36, 42].

Different diagnostic methods are available for specific identification of *Eimeria*. Traditional methods are based mainly on oocysts morphological characteristics under microscopic examination, parasite biology, clinical signs in the affected animals, and typical macroscopic lesions assessed by lesion scores [22]. However, due to the presence of intraspecies variation, the morphological method is not fully reliable since natural infections by *Eimeria* are generally mixed with more than one species and several species have confusing features [22, 30, 43]. Furthermore, morphological observations combined with faecal examination are very labor-intensive and require a skillful classification technique. Molecular techniques have been reported as useful for species identification or classification of this genus to overcome the limitations of traditional methods [5, 16, 45], and have further demonstrated the phylogenetic position of each *Eimeria* species and phylogenetic clades [46, 21, 24]. Molecular characterization of *Eimeria* species from goats has been reported from Australia [1], India [41], and Iran [17].

Livestock production, especially goat farming, is important for the livelihood of millions of rural people in Myanmar because it contributes to food security and the creation of assets. However, information on *Eimeria*, the causal agent of coccidiosis, one of the most economically important diseases in goats, has never been reported from Myanmar. The aim of this study was to demonstrate the presence of *Eimeria* in native goats from Myanmar for the first time and to describe the species of parasites using molecular and morphological tools.

## Materials and methods

### Study sites

The study design was cross-sectional and the study was conducted on traditional goat farms at three different villages within the Nay Pyi Taw area. Nay Pyi Taw is located between latitude 19°45′ N and longitude 96°60′ E with the following climate characteristics: altitude 115 m above sea level; annual rainfall 1167 mm; and annual temperature range 21.2–32.5 °C. Sampling period was between March and May, 2017. Measurement of oocyst sizes and molecular examinations were performed at the Laboratory of Parasitology, Faculty of Veterinary Medicine, Hokkaido University, Japan.

### Sample collection and processing

The study included 280 faecal samples (110 from males and 170 from females) collected from 12 traditional goat farms of indigenous breeds, Jade Ni and Htain San. Among the studied samples, 77 from kids under 4 months old, 110 from kids 4–12 months old, and 93 from animals older than 12 months, were examined for oocysts of *Eimeria*. The number of animals on each farm ranged between 10 and 80. Animals were kept in a semi-intensive management system: they were kept in wooden sheds with raised slatted floors at night and in the morning, and allowed to graze in the afternoon for 3–5 h on common pasture, on road sides or on uncultivated land. No feed supplementation was provided on any farm and there was no history of anticoccidial treatment against coccidiosis.

Faecal samples were collected directly from the rectum and stored on ice or in a refrigerator (4.0 °C) until microscopic examination. The presence of oocysts in the faecal samples was examined by the flotation method using Sheather’s sugar solution (SG = 1.26) as described by Salant et al. [34]. The washed faecal samples were then mixed with sugar solution and centrifuged at 2500 rpm for 10 min. The oocysts were then collected from the tops of the centrifuge tubes. Concentrated oocysts were identified on the basis of morphological characters. The *Eimeria* species were determined based on morphology of oocysts and sporocysts (shape, colour, form index, micropyle and polar cap, and presence or absence of residual body) [10]. A total of 10 morphologically characterized oocysts of each species was examined and photographed using microscopy. Then the length and width of 50 randomly selected sporulated oocysts of each sample were measured at 400× magnification using cellSens Imaging software (Olympus, Japan). Among the positive samples, 20 were randomly selected and oocysts per gram (OPG) were determined quantitatively by the modified McMaster method [23].

### DNA extraction

Oocyst samples were purified in several steps before DNA preparation. For each DNA preparation, approximately 500 morphologically similar oocysts were collected by using PicoPipet (Nepa Gene, Japan). Then the oocysts were washed three times with distilled water and finally concentrated to a volume of 50 μL. Before DNA extraction, the oocyst samples were transferred to a 1.5 mL centrifugal tube and five cycles of freezing/thawing were performed; quick freezing was performed in a −80 °C freezer for 5 min, while quick thawing was conducted in a 37 °C water bath for 5 min. Oocysts were crushed using 0.2 mm glass beads (Biomedical Science, Tokyo, Japan) followed by vortexing for 5 min at 2000 rpm [13]. Thereafter, DNA was extracted from the lysate using a PowerFecal^®^ DNA isolation kit (MO BIO Laboratories, USA), according to the manufacturer’s instructions. The DNA was quantified spectrophotometrically and stored at −20 °C for subsequent analysis.

### Identification of *Eimeria* species by PCR

The specific primers for both the cytochrome oxidase I (COI) and 18S rRNA genes were used based on the corresponding published sequences of related species. A partial mitochondrial COI gene sequence (723 bp) was amplified using a nested PCR, as described by Al-Habsi et al. [1]. A region of the 18S rRNA gene (630 bp) was amplified with the forward primer mCYC1FE 5′ – TACCCAATGAAAACAGTTT – 3′ and the reverse primer mCYC2RB 5′ – CAGGAGAAGCCAAGGTAGG – 3′ [15]. PCR was performed in a 25 μL volume containing 1 μL of DNA template, 0.5 μL of Tks Gflex DNA polymerase (1.25 U/μL) (TaKaRa Bio Inc., Shiga, Japan), 12.5 μL of 2× Gflex PCR Buffer (Mg^2+^, dNTP plus, TaKaRa Bio Inc.), and 10 μL of nuclease-free water (Promega, Madison, WI, USA). Thermocycling for both target genes was done with an initial denaturation step for 5 min at 94 °C, 45 cycles of denaturation for 30 s at 98 °C, annealing for 30 s at 55 °C, and extension for 2 min at 68 °C, and a final extension for 7 min at 68 °C. The second PCR was conducted with 1 μL of the first PCR amplification mixture as the template after 10-fold dilution in nuclease-free water. The PCR products were examined by 1% (for COI gene) and 2% (for 18S rRNA gene) Tris-acetate-EDTA (TAE) agarose gel electrophoresis, and stained with Red Safe Nucleic Acid Staining Solution (iNtRON Biotechnology Inc., Sungnum, Korea).

DNA fragments obtained from the PCR were excised from the gel and purified with a NucleoSpin^®^ Gel and PCR Clean-up Kit (MACHEREY-NAGEL, Düren, Germany), according to the manufacturer’s instructions, and submitted for direct sequencing using an Applied Biosystems 3130 Genetic Analyzer with a BigDye v3.1 Terminator cycle sequencing kit (Applied Biosystems, Inc., Carlsbad, CA, USA).

### Phylogenetic analysis

Multiple sequence alignment was performed using the sequence analysis software package ATGC version 7 (GENETYX Corporation, Tokyo, Japan). Further manual alignment and determinations of pairwise percentage of sequence identity were done by ClustalW. Phylogenetic relationships between sequences were assessed by using the maximum likelihood method in MEGA, version 6.06 [38]. A phylogenetic tree was constructed for *Eimeria* at the 18S locus with additional isolates from GenBank. Bootstrap analysis was done using 1000 replicates/tree. The obtained sequences were compared to those from the NCBI nucleotide database (http://www.ncbi.nlm.nih.gov/nuccore/). The nucleotide sequences for the analyzed COI and 18S rRNA genes of *Eimeria* were deposited in GenBank with accession numbers LC507792–LC507798 and LC508121–LC508123, respectively.

### Statistical analysis

Statistical analysis was performed using Epi Info™ software version 7.2 (https://www.cdc.gov/epiinfo/pc.html). Differences in coccidial occurrence among different age groups were analysed using Chi-square test and *p* < 0.05 was considered significant.

## Results

### Microscopic examination

Of 280 faecal samples examined, 168 (60.0%) were positive for *Eimeria* oocysts: 55 were from kids (71.4% of 77 kid samples), 61 from weaners (55.4% of 110 weaner samples) and 52 from adult goats (55.9% of 93 adult samples). The morphological features (shape and size) of the oocysts and sporocysts from the three identified *Eimeria* species were consistent with ranges previously reported for the respective species (Table 1). In 20 tested samples, OPG in two goats was >10,000, whereas OPG in 18 goats ranged from 100 to 6000. Three different *Eimeria* species were identified and their positive detection rates in the herd were: *E. arloingi* (25.4%), followed by *E. hirci* (20.7%), and *E. christenseni* (13.9%). The species detected and their positive rates over the age classes are given in Table 2. Among positive goats, the double infection rate with two *Eimeria* species was 22.6% (38/168) and the triple infection rate with three *Eimeria* species was only 2.4% (4/168). The frequency of multiple infections was higher in kids compared with other age classes (Table 3). Moreover, kids showed a higher risk for infection than other age groups (*χ*^2^ = 4.22955, *p* < 0.05). There was no significant association between sex and *Eimeria* infection (*χ*^2^ = 0.994652, *p* = 0.3186).

**Table 1:** Oocyst morphological features for *Eimeria* spp. from Myanmar goats (characterisation according to Eckert et al. [10]).

*Eimeria* spp.	Oocyst size mean (range) μm	Characteristics
*E. christenseni*	Height = 33.3 (30.5–35.6)	Ellipsoid-ovoid, bi-layered & thick wall, with micropolar cap, broadly elongated, ovoid sporocyst
	Width = 21.3 (18.1–25.9)	
*E. arloingi*	Height = 22.9 (21.2–23.0)	Ellipsoid-ovoid, bi-layered & thick wall, with micropolar cap, elongated ovoid sporocyst
	Width = 18.5 (17.2–20.1)	
*E. hirci*	Height = 20.03 (18.3–22.1)	Roundish oval, bi-layered wall, with micropolar cap, slightly ovoid-round sporocyst
	Width = 18.6 (16.1–21.3)	

**Table 2:** Percentage of infections in faecal samples for each *Eimeria* species in goats.

Species	No. of infected animals			Total no. infected (%)
	Kid (<4 mo)	Weaner (4–12 mo)	Adult (>12 mo)	
*E. christenseni*	10	10	15	39 (13.9)
*E. arloingi*	25	27	22	71 (25.4)
*E. hirci*	20	24	15	58 (20.7)

**Table 3:** The number of *Eimeria* species present in individual positive faecal samples.

Age class	No.	Positive (%)	No. of species in sample (%)		
			1	2	3
Kid < 4 mo	77	55 (71.4)*	46	9	0
Weaner 4–12 mo	110	61 (55.4)	45	13	3
Adult > 12 mo	93	52 (55.9)	35	16	1

* *χ*^2^ = 4.22955, *p* < 0.05.

### Phylogenetic analysis of the three *Eimeria* species based on the 18S rRNA and COI genes

**Figure 1:**
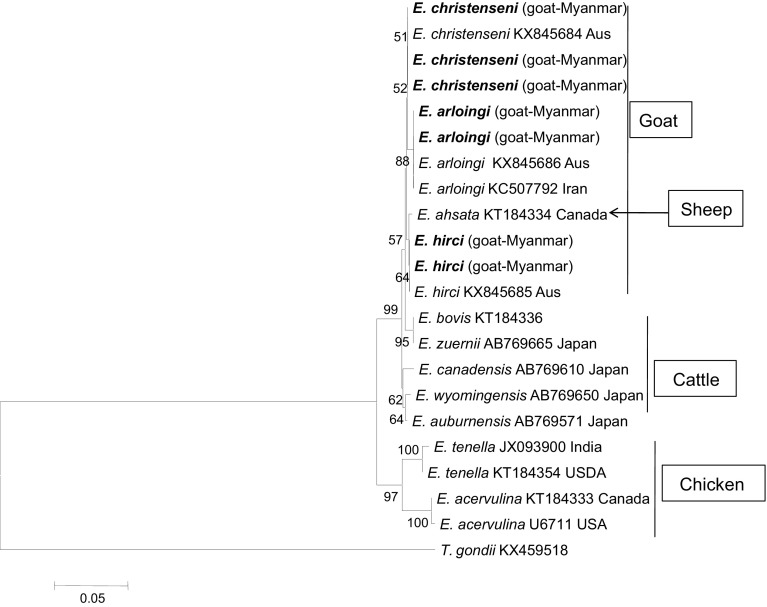
Phylogenetic tree of *Eimeria* species based on the 18S rRNA gene. The tree was built with the maximum likelihood method. Numbers indicate bootstrap percentages (1000 replicates). The scale indicates the divergence time.

Three 18S sequences obtained from this study showed 100% homology with *E. christenseni* (KX845684) reported from Australia and grouped in the same clade. Two sequences grouped in a clade with *E. arloingi* (KX845686 and KC507792) reported from Australia and Iran with 100% homology. Two sequences were 100% identical to *E. hirci* (KX845685) from Australia (Fig. 1).

COI sequences of *E. christenseni*, *E. hirci*, and *E. arloingi*, respectively, exhibited 98.9%, 98.4%, and 98.5% similarities with those reported from rangeland goats in Australia (KX857468, KX857469, and KX857470) (Fig. S1).

## Discussion

There are about 8.13 million goats in Myanmar [12], mostly reared for meat, although milk is also used by households in some areas [27]. More than half of the national goat population is found in the central region, the area studied. Economic losses due to coccidiosis have been reported by many researchers elsewhere [20]. The factors causing economic losses by coccidiosis in domestic animals include high mortality rates and lowered productivity due to poor growth, together with costs of anticoccidials, drug administration and disinfection [9]. In addition, in the case of moderate infection without clinical signs, these losses can be linked to reduced production (due to poor weight gain in particular) and infected animals appear more susceptible to other infections [6]. In two studies carried out in East Africa, coccidiosis appears to be a secondary cause of mortality amongst small ruminants in comparison with other parasitic or infectious diseases such as pneumonia or helminthiasis [19, 26].

In Myanmar, morphological and molecular data on *Eimeria* species in goats were not available. The present study has shown that a moderately high occurrence of *Eimeria* species (60.0%), particularly *E. arloingi* (52.7%), and *E. hirci* (43.0%), exists in Myanmar goats. Based on microscopic examination, studies on goats in dry tropical areas of Africa, such as in Senegal [40], Nigeria [44], and Zimbabwe [7], have shown high prevalence of *Eimeria* species infection, the common species being *E. arloingi* (64–80%) and *E. christenseni* (60%). Comparable studies from Asian countries such as Sri Lanka, Malaysia, India, and China also showed low to high prevalence of *E. arloingi* (21–73%) and *E. christenseni* (63–78%) [11, 14, 36, 42]. In contrast with other studies [42, 44], the occurrence of single-species infection was higher than mixed-species infection in this study. The reason might be the rearing system in our study area. The goats were reared in a semi-intensive management system, with average herd size ranging between 10 and 80, which is a smaller herd size than other reports [32]. According to a report by Tomczuk et al. [39], the dissemination of coccidia species is facilitated by rearing with high population density. As a result, the farming system in our study area might not favour the abundance and dissemination of *Eimeria* spp. In this study, we focused on the Nay Pyi Taw area only, and thus different species of *Eimeria* might be observed from goats in other parts of the country.

The current findings revealed that among the examined samples, 71.4% of 77 kids were found to be positive, which is a higher association with *Eimeria* infection (*p* < 0.05) than weaners and adults with positive rates of 55.4% and 55.9%, respectively. Frequency of infection was lower in adults probably due to more developed immunity in adults compared to kids [4]. These results are in agreement with previous reports by Balicka-Ramisz [3], Wang et al. [42], and Silva et al. [37].

Notwithstanding that *E. arloingi* and *E. christenseni* have been reported as pathogenic species, no clinical signs of coccidiosis were seen in any of the examined goats. Most samples collected from goats consisted of well-formed faeces and only a few had soft-formed faeces but severe diarrhoea was not seen in any of the animals examined. Some reports have shown the existence of genetic resistance to gastrointestinal parasites and coccidiosis in indigenous goats in Asia and Africa [8, 25, 28, 31]. In our study areas, all goats were allowed to graze in the afternoon for 3–5 h on common pasture shared with other goats in the same village. Pasture in the study area was mostly grazed by goats and cattle. Since *Eimeria* species are host-specific, infection cannot be transmitted from other animal species. The parasites seemed to be circulating among goats grazing this area and the goats might become chronic carriers. The infected goats without clinical symptoms in a herd retain their infections year-round, continually contaminating the environment with oocysts. Therefore, the infected goats serve as a source of re-infection and of new infections for young kids [2].

Diagnosis of *Eimeria* species in goats mainly relies on morphological examination. In this study, to support our microscopic identifications, findings were substantiated using molecular techniques based on PCR amplifying the parasite 18S rRNA and COI genes. The 18S rRNA gene has been widely used for molecular detection of *Eimeria* species; however, since this gene is highly conserved, it is not appropriate to evaluate genetic diversity among *Eimeria* species. The phylogenetic tree generated in this study suggests a shared evolutionary history for *Eimeria* in cattle (*E. bovis* and *E. zuernii*), sheep (*E. ahsata*) and goats, and therefore they may have evolved from one common ancestor. In addition, high similarity of 18S rDNA sequences between *E. arloingi* and *E. ahsata* has also been reported from Iran [17]. Though the COI gene has been suggested as a suitable target to differentiate closely related *Eimeria* species in small ruminants [1], the number of sequences available in GenBank is still limited. We can compare COI sequences reported from rangeland goats in Australia only. Thus, the sequence data obtained in the present study will make a contribution to understand the genetic diversity and geographic distribution of *Eimeria* species infecting small ruminants worldwide.

In terms of poverty alleviation in Myanmar, it is important to maintain and develop goat-related industries. Proper management should be practiced to prevent losses and reduced productivity from coccidiosis in young animals by: reducing the level of environmental contamination by infectious oocysts; minimizing stress; and avoiding crowding of kids [2, 6]. As in other regions, the significance of coccidiosis still seems to be underestimated by both veterinarians and farmers. It is necessary to be aware of the problem and practice control strategies such as maintaining hygienic conditions and using anticoccidial drugs.

In conclusion, this is the first report of *Eimeria* species from goats in Myanmar. A further innovation in Myanmar was that identification was undertaken by morphological determination and confirmed by molecular analysis. Further research is needed to elucidate the prevalence of *Eimeria* infection in goats and its relationship with the management system, and seasonal and geographical variations for the whole country. In addition, quantitative analysis for *Eimeria* infection should also be considered and its economic impact in future studies. This report provides occurrence and molecular information on the *Eimeria* species in Myanmar goats that could be used for future molecular investigations.

## Conflict of interest

The authors declare that they have no competing interests.

## Supplementary Materials

**Figure S1.**
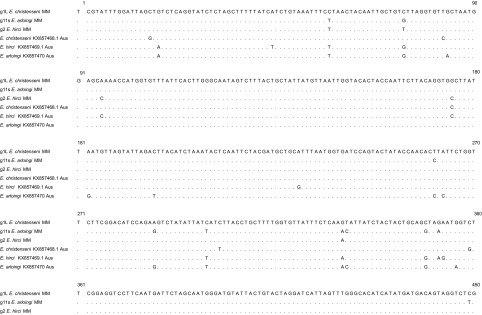
Alignment of the COI partial sequences isolated in this study with those of *E. christenseni*, *E. arloingi*, and *E. hirci* reported in GenBank. Dots indicate bases that are identical to the reported data.Supplementary material is available at https://www.parasite-journal.org/10.1051/parasite/2020037/olm
